# The Transcriptional Complex Sp1/KMT2A by Up-Regulating Restrictive Element 1 Silencing Transcription Factor Accelerates Methylmercury-Induced Cell Death in Motor Neuron-Like NSC34 Cells Overexpressing SOD1-G93A

**DOI:** 10.3389/fnins.2021.771580

**Published:** 2021-11-26

**Authors:** Natascia Guida, Luca Sanguigno, Luigi Mascolo, Lucrezia Calabrese, Angelo Serani, Pasquale Molinaro, C. Geoffrey Lau, Lucio Annunziato, Luigi Formisano

**Affiliations:** ^1^IRCCS SDN, Naples, Italy; ^2^Division of Pharmacology, Department of Neuroscience, Reproductive and Dentistry Sciences, School of Medicine, “Federico II” University of Naples, Naples, Italy; ^3^Department of Neuroscience, City University of Hong Kong, Hong Kong, China

**Keywords:** MeHg, SOD1-G93A, Sp transcription factors, REST (RE-1 silencing transcription factor), motor neuronal cell death

## Abstract

Methylmercury (MeHg) exposure has been related to amyotrophic lateral sclerosis (ALS) pathogenesis and molecular mechanisms of its neurotoxicity has been associated to an overexpression of the Restrictive Element 1 Silencing Transcription factor (REST). Herein, we evaluated the possibility that MeHg could accelerate neuronal death of the motor neuron-like NSC34 cells transiently overexpressing the human Cu^2+^/Zn^2+^superoxide dismutase 1 (SOD1) gene mutated at glycine 93 (SOD1-G93A). Indeed, SOD1-G93A cells exposed to 100 nM MeHg for 24 h showed a reduction in cell viability, as compared to cells transfected with empty vector or with unmutated SOD1 construct. Interestingly, cell survival reduction in SOD1-G93A cells was associated with an increase of REST mRNA and protein levels. Furthermore, MeHg increased the expression of the transcriptional factor Sp1 and promoted its binding to REST gene promoter sequence. Notably, Sp1 knockdown reverted MeHg-induced REST increase. Co-immunoprecipitation experiments demonstrated that Sp1 physically interacted with the epigenetic writer Lysine-Methyltransferase-2A (KMT2A). Moreover, knocking-down of KMT2A reduced MeHg-induced REST mRNA and protein increase in SOD1-G93A cells. Finally, we found that MeHg-induced REST up-regulation triggered necropoptotic cell death, monitored by RIPK1 increased protein expression. Interestingly, REST knockdown or treatment with the necroptosis inhibitor Necrostatin-1 (Nec) decelerated MeH-induced cell death in SOD1-G93A cells. Collectively, this study demonstrated that MeHg hastens necroptotic cell death in SOD1-G93A cells *via* Sp1/KMT2A complex, that by epigenetic mechanisms increases REST gene expression.

## Introduction

Exposure to the environmental methylmercury has been identified as a risk to the development of several neurodegenerative diseases, including Alzheimer’s disease, Parkinson’s disease (Chin-Chan et al., 2015), stroke (Chen C. et al., 2018) and amyotrophic lateral sclerosis (ALS) (Bailey et al., 2017). ALS is a motor neuron disease associated with failure movements of the voluntary muscles and the typical time from symptom onset to death is 2–3 years (Westeneng et al., 2018). Familial and sporadic forms of ALS share common cellular alterations, such as neuromuscular junction (NMJ) disruption and loss of motor neurons (MNs) in the spinal cord, brainstem and cortex (Ito et al., 2016). Focusing on the familial ALS forms, one of the most investigated genetic mechanism is the mutation of glycine 93 in alanine (G93A) in the Cu/Zn Superoxide Dismutase-1 (SOD1) gene (Chen et al., 2013), whereas in sporadic forms the Transactive Response DNA binding protein (TDP-43) has been identified as the major pathological molecular determinant (Halliday et al., 2016). Interestingly, the transgenic mice overexpressing the human mutated SOD1 gene or the TDP-43 show significant neurodegeneration and symptoms similar to ALS in human patients (Philips and Rothstein, 2015). Our previous reports demonstrated that Restrictive Element 1 Silencing Transcription factor (REST) up-regulation is involved in the molecular mechanisms by which the environmental neurotoxicants MeHg and polychlorinated biphenyl (PCBs) induce apoptotic and necroptotic cell death in neuronal cells, respectively (Guida et al., 2017b). Notably, necroptosis is activated indirectly by the transcription factor REST *via* its binding to the promoter sequence of caspase-8 gene, whose down-regulation is essential for the activation of necroptotic pathway (Guida et al., 2017b). Moreover, MeHg exposure increases the binding of the transcriptional activator JunD on REST promoter sequence and causes its consequent transcriptional activation in neuronal cells (Guida et al., 2018). REST promoter sequence is composed by three alternative promoters A, B, and C, that regulate the exons a, b and c, respectively. Interestingly, each exon can splice directly into exon d, which contains the ATG translation initiation site of REST sequence (Koenigsberger et al., 2000). To our knowledge REST is transcriptionally activated by the following proteins: Specificity protein 1 and 3 (Sp1, Sp3), JunD and cAMP response element-binding protein (CREB) (Kreisler et al., 2010; Ravache et al., 2010; Guida et al., 2015a). It is well known that the transcriptional factors activate gene transcription by recruiting epigenetic modifiers, such as histone acetyl transferases (HATs) and histone methyl transferases (HMTs) on the promoters of their target genes (Gardner et al., 2011). Importantly, Sp1 and CREB, but not JunD and Sp3, physically interact with the epigenetic writer HMT chromatin Lysine (K)-specific methyltransferase 2A (KMT2A) (Ernst et al., 2001; Xu et al., 2017), that is the enzyme catalyzing the reaction of trimethylation of the lysine 4 on the histone protein 3 (H3K4-me3), a critical marker of active transcription (Yang and Ernst, 2017). It is noteworthy that the KMT2A overexpression and the consequent increase of H3K4-me3 has been related to cell death in cells exposed to the apoptotic inducer tunycamicin (Wang et al., 2014) and after stroke (Feng et al., 2020).

Since exposure to MeHg: (1) hastens the onset of amyotrophic lateral sclerosis-like phenotype in SOD1-G93A mice by activating glutamate receptors (Johnson et al., 2011) and (2) determines neuronal cell death by increasing REST expression (Guida et al., 2017a,^2018^), we investigated the possible role of MeHg to accelerate death in NSC34 motor neuron-like cells transiently transfected with SOD1-G93A construct, *via* REST up-regulation. Furthermore, we studied the transcriptional and epigenetic mechanisms increasing REST expression *via* its well-known transcriptional modulators Sp1, Sp3, CREB and JunD. Finally, since REST overexpression in neurons determines cell death by activating necroptosis, we verified whether MeHg *via* REST could activate necroptosis in motor neuron-like SOD1-G93A NSC34 cells.

## Materials and Methods

### Material, Cell Cultures, and Drug Treatment

The NSC34 cells were purchased by Tebu-BIO [Magenta, (MI) Italy]. Cells were used between 5–20 passages. Cells were cultured in Dulbecco’s Modified Eagle Medium (DMEM) in the presence of 10% inactivated fetal bovine serum and penicillin/streptomycin at 37°C, 5% CO2 and 95% air, in a cell culture incubator. SH-SY5Y cells were purchased and grown as previously published (Guida et al., 2015a). Methylmercury (II) chloride (MeHg) (cod: 442534 stock solution 100 mM) and Necrostatin-1 (Nec) (cod. N9037; stock solution 20 mM) were purchased from Sigma both obtained from Sigma–Aldrich (St. Louis, MO, United States) and were dissolved as previously reported (Guida et al., 2018). Culture media and sera were purchased from Invitrogen (Milan, Italy). All chemicals were diluted in cell culture medium. Synthetic oligonucleotides were from Eurofins Genomics. For Nec experiments NSC34 cells were pre-incubated for 2 h in full medium with the drug at 1, 5, 10, and 20 μM, followed with MeHg 100 nM for 24 h. For MTT and LDH assays, cells were plated in 24 well plates at a density of 1 × 10^4^ cells/well; for qRT-PCR, they were plated in 60 mm plates at a density of 5 × 10^4^ cells/plate; for Western blot, Immunoprecipitation and ChIP, they were plated in 100 mm plates at 6 × 10^6^ cells/plate.

### Cell Transfection

siRNA for REST (siREST) (EMU029201), siRNA for Sp1 (siSp1) (EMU061231) and siRNA for KMT2A (siKMT2A) (EMU180041) were purchased from Sigma-Aldrich, whereas the negative control (siCTL) (001910-10-05) was purchased from Dharmacon. NSC34 at 60% confluence were transfected with siRNAs (25 nM) by using Lipofectamine 2000 in Optimem medium, according to the protocol by the manufacturer (Invitrogen Srl, S. Giuliano Milanese Italy). For constructs transfection in NSC34 cells have been used the following plasmids: (1) pcDNA3.1, (2) pF151 pcDNA3.1(+)SOD1WT and (3) pF155 pcDNA3.1(+)SOD1-G93A, that were a gift from Elizabeth Fisher (Addgene plasmid # 26397 and # 26401) (Stevens et al., 2010). The amount of DNA constructs used for transient transfection (60% confluence) was: 500 ng DNA in 24-multiwell plates for MTT and LDH assays, 10 μg of DNA in 100 mm dishes for quantitative real time PCR (Q-PCR), Western blot and ChIP analysis. 24 h after transfection low serum medium containing vehicle or 100 nM MeHg was added. Transfection efficiency was evaluated by western blot analysis to demonstrate the presence of the human SOD1 protein and by Q-PCR to assess the levels of REST, Sp1 and KMT2A mRNAs after siRNAs treatment (Chong et al., 2021).

### Cell Viability Assessment

Cellular vitality is related to mitochondrial function which was assessed by measuring the activity of mitochondrial dehydrogenases. The soluble compound 3-(4,5-dimethylthiazol-2-yl)-2,5-diphenyltetrazolium bromide (MTT) was used as substrate. Mitochondrial dehydrogenases reduce MTT by converting it into formazan, a salt that is insoluble in water and optically active at 540 nm. The amount of formazan produced is directly proportional to the metabolic activity of mitochondrial dehydrogenases and therefore, indirectly, to cell viability. At the end of the exposure to MeHg, the culture medium was removed and the cells were incubated in 2 ml of a PBS solution containing MTT at a concentration of 0.5 mg/ml for 1 h at 37°C in an atmosphere consisting of 5% CO2 and 95% air. At the end of the hour, the incubation was interrupted by removing the solution and adding 1 ml of dimethyl sulfoxide (DMSO) to solubilize the formed formazan salts. The results were obtained by spectrophotometric analysis and were expressed as the percentage of survival of cells treated with MeHg compared to the values obtained from cultures exposed to the vehicle (Veh) to which the 100% survival value was attributed.

### Determination of Cell Death

Cell death was evaluated with the lactate dehydrogenase (LDH) cytotoxicity kit from Cayman, as previously reported (Guida et al., 2018). NSC34 cells have been pre-treated for 2 h with Nec at 1, 5, 10, and 20 μM or with vehicle, then they have been exposed to MeHg 100 nM for 24 h. Cells treated with 1% Triton X-100 (Sigma) were used as positive control of cell death.

### Quantitative Real Time PCR

Total RNA was extracted using Trizol according to the manufacturer’s instructions and quantified spectrophotometrically. The retro-transcription was performed as previously reported (Formisano et al., 2014). The reaction mixture was incubated for 60 min at 42°C and 2 min at 94°C, followed by 35 cycles of 30 s at 95°C, 30 s at 60°C and 30 s at 72°C. Normalization of the results was performed by β-Actin (Actin). The oligonucleotide sequences were: REST (m): (FW 5′-GTTCAGCACGTGCGAACTCA-3′ and RV 5′-TCCGCATGTGTCGCGTTA-3′); for Sp1 (m): (FW 5′-GCCTGTTAGGAGGTCCCTGAA-3′ and RV 5′-AGGCTGCCCATTTGTACTCATTTA-3′); for Sp3 (m): (FW 5′-CCTGACACAGACCCCTGTTGA-3′ and RV 5′-CAGTG CTCGCATCTGTGGAA-3′); for KMT2A (m): (FW 5′-GACCGAATCGGACTAAACACTTTC-3′ and RV 5′-GTGACCTTGCCTAGTAATCGAACTT-3′); for CREB (m): (FW 5′-TACTTCTCCATTCTCTCCTTCTCA-3′ and RV 5′-TGTAC GCGAGCGAGAGG-3′), for JunD (m): (FW 5′-GCTCTCT CTCCTCCCGACA-3′ and RV 5′-GGCGAACCAAGGATTAC GGA-3′) for β-Actin (m): (FW 5′-GTCGTACCACTG GCATTGTG-3′ and RV 5′-AGAGGGTAATCGTGCGTGAC-3′). Changes in gene expression between groups were represented as the mean of the relative quantification (RQ) values, that was calculated as the difference in threshold cycle (ΔCt) between the target gene and the reference gene (2^–ΔΔCT^ = RQ).

### Western Blotting and Immunoprecipitation

Protein expression was evaluated using standard Western Blotting methods. After the treatments, the cells were collected and centrifuged for 30 min at 14,000 RPM. The cells were washed only once with cold PBS, then a lysis buffer was added: Tris–HCl pH 7.5 (1 M), NaF (0.5 M), NaCl (4 M), PMSF (100 mM), Sodium Vanadate (100 mM), NP-40 (20%) plus protease inhibitor cocktail. The determination of the protein content of the samples was carried out according to the Bradford method. Aliquots containing 80 μg of proteins were fractionated by polyacrylamide gel electrophoresis (BIORAD Laboratories, Milan, Italy) in SDS (0.1%) (SDS-PAGE) and the separated proteins were transferred to a nitrocellulose filter. The filters were blocked for 2 h at room temperature in a blocking solution prepared with 0.1% Tween-20 (FlukaChemie GmbH, Italy), in 5% casein (Bio-Rad Laboratories, Milan, Italy), dissolved in TBS tween (Bio-Rad Laboratories, Milan, Italy).

**FIGURE 1:**
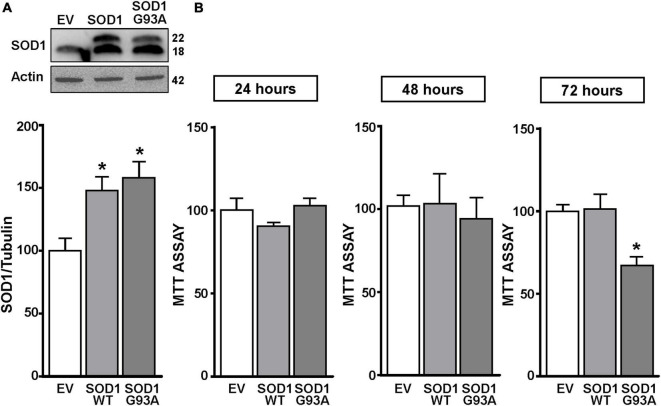
SOD1 protein levels and cell viability in motor neuron like NSC34 cells transiently transfected with the empty vector, SOD1-WT and SOD1-G93A constructs. **(A)** Western Blot of SOD1 in NSC34 transiently transfected with the followed vectors: (1) empty vector (EV), (2) wild type human SOD1 (SOD1-WT) and (3) human SOD1 containing the G93A mutation (SOD1-G93A). Graphs show quantification of ratio of SOD1 and Actin. Bars represent mean ± SD (*n* = 3); *p ≤ 0.05 versus EV. **(B)** Effects of EV, SOD1-WT and SOD1-G93A vectors on cell survival, measured by MTT assay after 24, 48, and 72 h transfection. Bars represent mean ± SD (*n* = 3); *p ≤ 0.05 versus EV and SOD1-WT.

Immunoprecipitation of KMT2A in MeHg-treated SOD1-G93A cells was performed as previously described (Guida et al., 2015b). Briefly, cell lysates (1 mg) were immunoprecipitated overnight at 4°C using KMT2A antibody (3 μg) and IgG as negative control. Protein A/G-agarose beads (25 μl) (sc-2003 Santa Cruz Biotechnology) were used to precipitate the bound protein. The antibodies used were: anti-REST (07-579, Millipore, 1:1,000); anti-Sp1 (sc-14027, Santa Cruz Biotechnology, 1:500); anti-KMT2A (sc-374392, Santa Cruz Biotechnology, 1:500); H3K4-3me (PA5-17420, Invitrogen, 1:1,000); anti-caspase-8 (sc-6136, Santa Cruz Biotechnology, 1;1,000); RIPK1 (sc-41169, Santa Cruz Biotechnology, 1:500); anti-SOD1 (cod: PA5-27240, Thermo Fischer Scientific, 1:1,000) and anti-Tubulin (T5168, Sigma-Aldrich, 1:10,000). Total lysates of differentiated SH-SY5Y cells, that basally express REST, Sp1; KMT2A, H3K4-me3, RIPK1 and caspase-8 (Das et al., 2009; Guida et al., 2015a, 2017b; Wang et al., 2019) were used as positive control for all antibodies used for Western Blot experiments (Supplementary Figure 3). Total lysate of NSC34 cell transfected with SOD1-G93A construct is the positive control for SOD1 antibody (Figure 1A).

The protein bands under examination were visualized using a chemoluminescence system (ECL Western Detection Kit; Amersham, Arlington Heights, IL, United States). The bands were quantized by densitometry. Normalization of the results was ensured by hybridizing the blots with anti-tubulin antibody.

### Chromatin Immunoprecipitation

Experiments were performed as previously reported (Guida et al., 2018). Briefly, NSC34 cells were cross-linked with 1% formaldehyde and then the reaction was stopped by adding glycine 0.125 M. Samples were washed four times in cold PBS containing anti-protease cocktail and lysis buffer. Chromatin was fragmented into 200–500 bp by sonication. Cells chromatin lysates were incubated overnight with 5 μg of antibody for Sp1 (sc-14027) and KMT2A (sc-374392) from Santa Cruz Biotechnology, H3K4-me3 (PA5-17420, Thermo Fisher Scientific), and RNA Pol II (05-623, Merck Millipore). Normal rabbit IgG (sc-2357, Santa Cruz Biotechnology) was used as negative control. After immunoprecipitation, the DNA-histone complex was collected with 50 μl of salmon sperm DNA/protein A or G-agarose beads for 2 h (16-157, 16-201, Merck Millipore) and subsequently processed as previously reported (Guida et al., 2018). The oligonucleotides used for the amplification of immunoprecipitated DNA recognizing A region of REST mouse promoter sequence were already published (Ravache et al., 2010). Binding activity was normalized for the total input of chromatin and graphically represented as percentage of vehicle.

### Statistical Analysis

All experiments were performed in triplicate. The data were represented as means ± SD. For the analysis an unpaired Student’s *t* test for statistic between two experimental groups and the one-way ANOVA with Tukey’s *post hoc* test for more than two experimental conditions were used.

## Results

### MeHg-Induced Cell Death *via* Restrictive Element 1 Silencing Transcription Factor Started From 24 h in Motor Neuron-Like SOD1-G93A Cells and Only After 72 h in Empty Vector and SOD1-WT Cells

**FIGURE 2:**
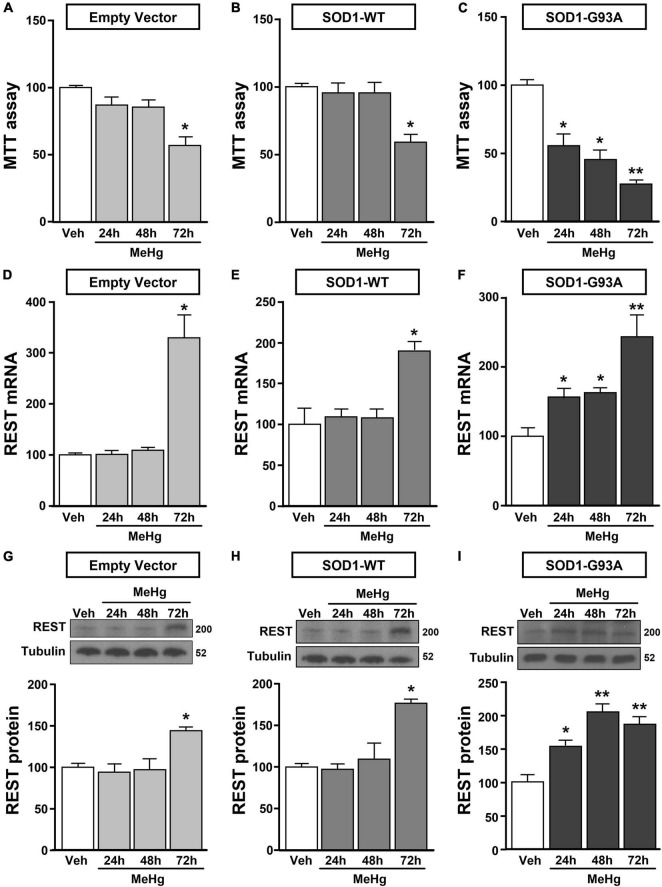
Effect of MeHg (100 nM) at 24, 48 and 72 h on survival and on REST mRNA and protein levels in motor neuron-like NSC34 EV, SOD1-WT and SOD1-G93Acells. **(A–C)** Effect of 24, 48, and 72 h of MeHg (100 nM) exposure on cell viability in: **(A)** EV, **(B)** SOD1-WT and **(C)** SOD1-G93A cells. Bars represent mean ± SD (*n* = 3); *p ≤ 0.05 versus vehicle (Veh); **p ≤ 0.05 versus all. **(D–I)** Effect of 24, 48, and 72 h of MeHg (100 nM) exposure on REST mRNA and protein levels in: **(D,G)** EV, **(E,H)** SOD1-WT and **(F,I)** SOD1-G93A cells. Bars represent mean ± SD (*n* = 3). *p ≤ 0.05 versus vehicle (Veh), **p ≤ 0.05 versus all.

**FIGURE 3:**
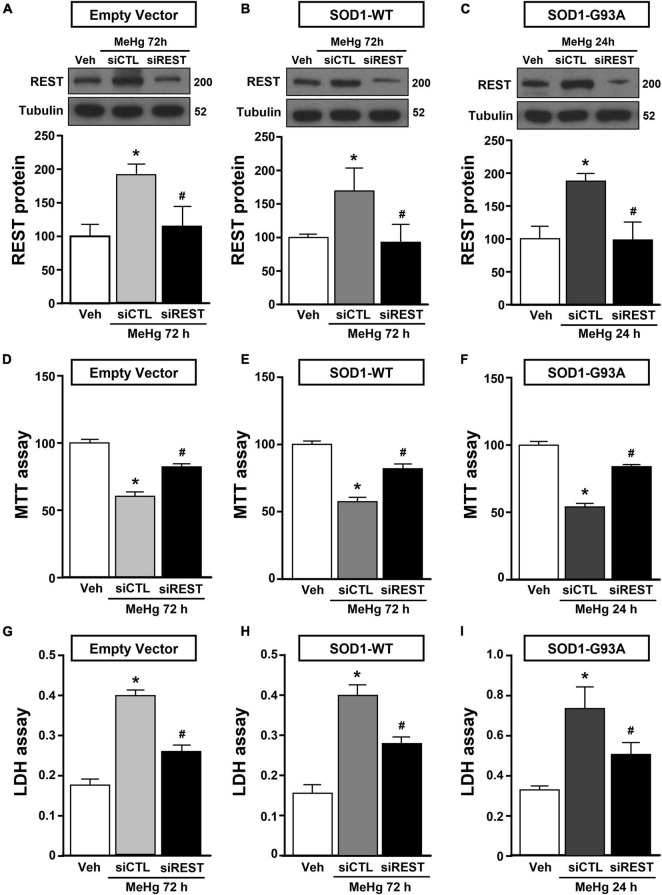
Effect of REST knocking-down to counteract MeHg-induced cell death in SOD1-G93A motor neuron like NSC34 cells. **(A,B,D,E,G,H)** Effect of siRNA for REST (siREST) on REST protein levels, cell viability and LDH release in: **(A,D,G)** EV, and **(B,E,H)** SOD1-WT exposed to 72 h of MeHg (100 nM). Bars represent mean ± SD (*n* = 3). *p ≤ 0.05 versus vehicle (Veh), ^#^*p* ≤ 0.05 versus siCTL + MeHg. **(C,F,I)** Effect of siRNA for REST (siREST) on REST protein levels, cell viability and LDH release in SOD1-G93A exposed to 24 h of MeHg (100 nM). Bars represent mean ± SD (*n* = 3). *p ≤ 0.05 versus vehicle (Veh), ^#^*p* ≤ 0.05 versus siCTL + MeHg.

NSC34 cells were transiently transfected separately with the following plasmids: (1) empty vector (EV), (2) the vector overexpressing the wild type SOD1 gene (SOD1-WT) and (3) the vector overexpressing the G93A mutant SOD1 gene (SOD1-G93A). As shown in Figure 1A, SOD1-WT and SOD1-G93A cells showed 48 and 58% increase in SOD1 protein expression, respectively, compared to EV group, thus confirming the transfection efficiency. As expected, cell survival significantly decreased after 72 h transfection in SOD1-G93A cells, but not in SOD1-WT and EV cells, as revealed by MTT (Figure 1B) and LDH assays (Supplementary Figures 2A–C). To study the possible role of G93A mutation in accelerating MeHg-induced cell death, EV, SOD1-WT and SOD1-G93A cells were exposed for 24, 48, and 72 h to 100 nM of MeHg. This concentration (100 nM) of MeHg was used because it reduced cell viability after 72 h, but not at 24 and 48 h, in NSC34 motor neuron-like cells transfected with EV (Supplementary Figures 1A–C). Notably, SOD1-G93A experimental group showed a significant reduction in cell vitality already after 24 h of 100 nM MeHg exposures. By contrast, in EV and SOD1-WT cells a significant reduction in cell survival was observed only after 72 h of MeHg exposure, as revealed by MTT (Figures 2A–C) and LDH assays (Supplementary Figures 2D–F). Since 100 nM MeHg treatment for 24 h damaged approximately 50% of motor neuron-like NSC34 SOD1-G93A cells, this concentration and this time point were chosen for our experiments. In accordance with MeHg-induced cell death, REST mRNA and protein expression increased at 24, 48 and 72 h in SOD1-G93A cells, whereas in EV and SOD1-WT cells REST mRNA and protein increased only after 72 h of MeHg treatment (Figures 2D–I). These results suggest that SOD1-G93A cells are more sensitive to MeHg-induced neurotoxic effect *via* REST up-regulation than SOD1-WT cells. In support to this result, REST was knocked-down by a specific siRNA (siREST) transfection that reduced its mRNA level of 70% compared to siCTL (Supplementary Figure 3). Indeed, siREST reverted MeHg-induced REST protein increase (Figures 3A–C) and prevented SOD1-G93A cell death at 24 h, whereas this effect was exerted at 72 h in EV and SOD1-WT cells (Figures 3D–I).

### MeHg-Induced Restrictive Element 1 Silencing Transcription Factor mRNA and Protein Increase Was Determined by Sp1 Up-Regulation in SOD1-G93A Motor Neuron-Like Cells

**FIGURE 4:**
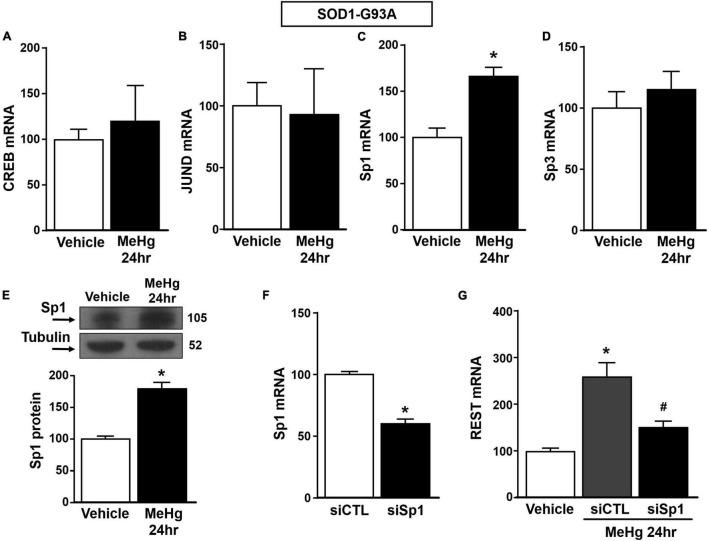
Effect of Sp1 knocking-down on REST gene in SOD1-G93A motor neuron-like NSC34 cells exposed to MeHg (100 nM) at 24 h. **(A–D)** qRT-PCR of CREB, JUND, Sp1 and Sp3 genes in SOD1-G93A cells exposed for 24 h to MeHg (100 nM). Graph shows the quantification of the ratio of CREB, JUND, Sp1 and Sp3 to β-Actin. Bars represent mean ± SD (*n* = 3). *p ≤ 0.05 versus Veh. **(E)** Western Blot of Sp1 in SOD1-G93A cells treated for 24 h with MeHg. Graph shows the quantification of the ratio of Sp1 to Tubulin. Bars represent mean ± SD (*n* = 3). *p ≤ 0.05 versus Veh. **(F)** qRT-PCR of Sp1 in SOD1-G93A cells treated with siCTL or siSp1. Graph shows the quantification of the ratio of Sp1 to β-Actin. Bars represent mean ± SD (*n* = 3). *p ≤ 0.05 versus siCTL. **(G)** qRT-PCR of REST in MeHg-exposed SOD1-G93A cells transfected with siSp1. Graph shows the quantification of the ratio of REST to β-Actin. Bars represent mean ± SD (*n* = 3). *p ≤ 0.05 versus Veh, ^#^*p* ≤ 0.05 versus siSp1 + MeHg 24 h.

Since it has been reported that REST gene is transcriptionally modulated by the two Sp family transcription factors Sp1 and Sp3, CREB, and JunD (Guida et al., 2015a, 2017b), we evaluated the effect of MeHg exposure to modulate the mRNA levels of the above mentioned transcriptional factors in SOD1-G93A cells. We found that 24 h exposure to 100 nM MeHg induced an increase in Sp1, but not in CREB, JunD and Sp3 mRNA expression (Figures 4A–D). Coherently, 24 h of MeHg exposure determined a significant increase also in Sp1 protein expression (Figure 4E). In order to clarify the possible role of Sp1 in the modulation of REST gene in MeHg-induced toxicity in SOD1-G93A cells, Sp1 was silenced by transfection with a specific siRNA (siSp1). The transfection efficiency was demonstrated by 40% reduction of Sp1 mRNA levels (Figure 4F). Notably, siSp1 counteracted the effect of MeHg on REST mRNA increase (Figure 4G).

### Sp1 by Forming a Complex With the Histone Lysine Methyltransferase KMT2A Up-Regulated Restrictive Element 1 Silencing Transcription Factor in SOD1-G93A Motor Neuron-Like Cells

**FIGURE 5:**
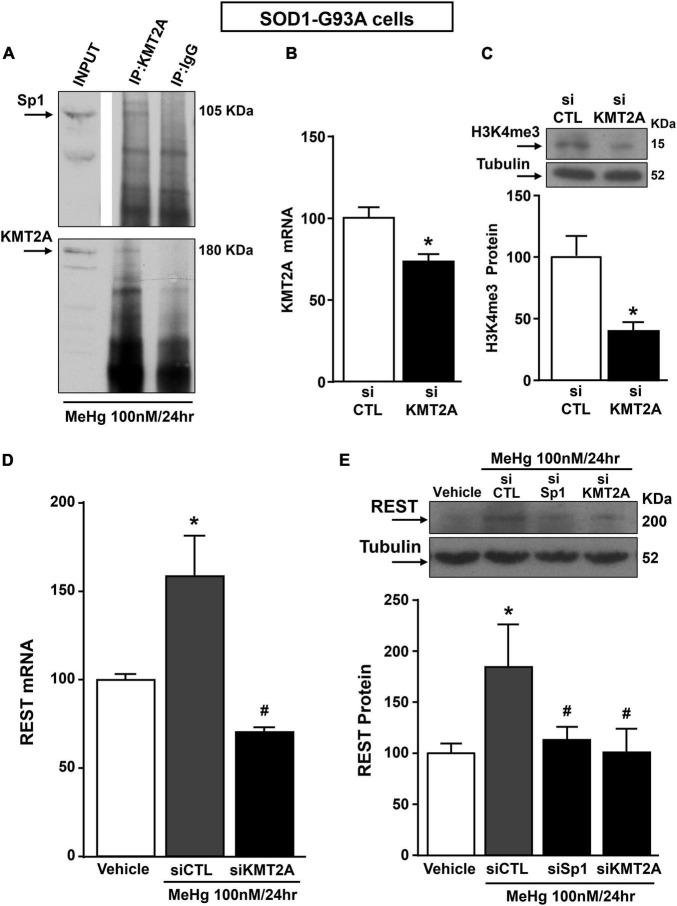
Sp1 increased REST gene levels in SOD1-G93A motor neuron-like NSC34 cells exposed for 24 h to MeHg (100 nM) *via* KMT2A recruitment. **(A)** Representative Western Blotting showing co-immunoprecipitation between KMT2A and Sp1. **(B)** qRT-PCR of KMT2A in SOD1-G93A cells transfected with siKMT2A. Graph shows the quantification of the ratio of KMT2A to β-Actin. Bars represent mean ± SD (*n* = 3). *p ≤ 0.05 versus siCTL. **(C)** Representative Western Blotting with quantification of trimethylated-H3K4 (H3K4-me3) in SOD1-G93A cells transfected with siCTL and siKMT2A. Graph shows the quantification of the ratio of H3K4-me3 to Tubulin. Bars represent mean ± SD (*n* = 3). *p ≤ 0.05 versus siCTL. **(D)** qRT-PCR of REST in MeHg-exposed SOD1-G93A cells transfected with siKMT2A. Graph shows the quantification of the ratio of REST to β-Actin. Bars represent mean ± SD (*n* = 3). *p ≤ 0.05 versus Veh, #*p* ≤ 0.05 versus siCTL + MeHg 24 h. **(E)** Western Blot of REST in MeHg-exposed SOD1-G93A cells transfected with siSp1 and siKMT2A. Graph shows the quantification of the ratio of REST to Tubulin. Bars represent mean ± SD (*n* = 3). *p ≤ 0.05 versus Veh, ^#^*p* ≤ 0.05 versus siCTL + MeHg 24 h.

**FIGURE 6:**
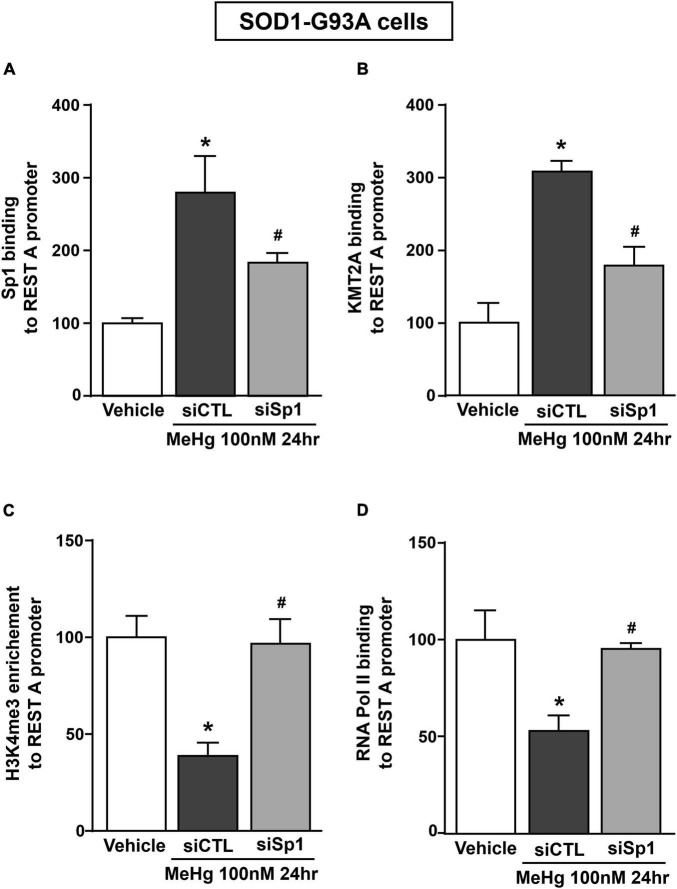
siSp1 reduced Sp1, KMT2A, H3K4-me3 and RNA-Pol II binding on REST A mouse promoter sequence in SOD1-G93A motor neuron-like NSC34 cells exposed to MeHg (100 nM). **(A–D)** ChIP analysis with antibodies for: **(A)** Sp1, **(B)** KMT2A, **(C)** H3K4-me3 and **(D)** RNA-Pol II of REST A mouse promoter sequence exposed to MeHg 100 nM in SOD1-G93A cells transfected with siSp1. Binding activity is graphically represented as the percentage of vehicle. Bars represent mean ± SD (*n* = 3). *p ≤ 0.05 versus Vehicle, **^#^***p* ≤ 0.05 versus siCTL + MeHg 100 nM/24 h.

Recently it has been demonstrated that Sp1 interacts with the epigenetic enzyme KMT2A in the breast cancer cell line MCF7 cells (Xu et al., 2017). The coimmunoprecipitation assay performed in SOD1-G93A cells exposed for 24 h to 100 nM MeHg revealed that Sp1 physically interacted with KMT2A (Figure 5A upper panel). The presence of KMT2A protein in the immunoprecipitated pool has been validated by immunoblot with KMT2A antibody (Figure 5A upper panel). On the basis of the interaction between Sp1 and KMT2A we examined the possibility that KMT2A could modulate REST expression in SOD1-G93A cells. Silencing of KMT2A (siKMT2A) significantly reduced its mRNA expression of 37% and the trimethylation of the lysine-4 on the histone-3 (H3K4-me3) protein levels of 60% (Figures 5B,C), thus confirming the transfection efficiency. It is noteworthy that siKMT2A counteracted MeHg-induced REST mRNA and protein up-regulation (Figures 5D,E). Interestingly, Chromatin Immunoprecipitation (ChIP) assay revealed that in MeHg-treated SOD1-G93A cells siSp1 reduced the Sp1 and KMT2A binding on REST A promoter region, by inducing a reduction of two well-known markers of gene transcription H3K4-me3 and RNA polymerase II (RNA-Pol II) (Figures 6A–D).

### Restrictive Element 1 Silencing Transcription Factor Knocking-Down Prevented MeHg-Induced Necroptotic Cell Death in SOD1-G93A Motor Neuron-Like Cells

**FIGURE 7:**
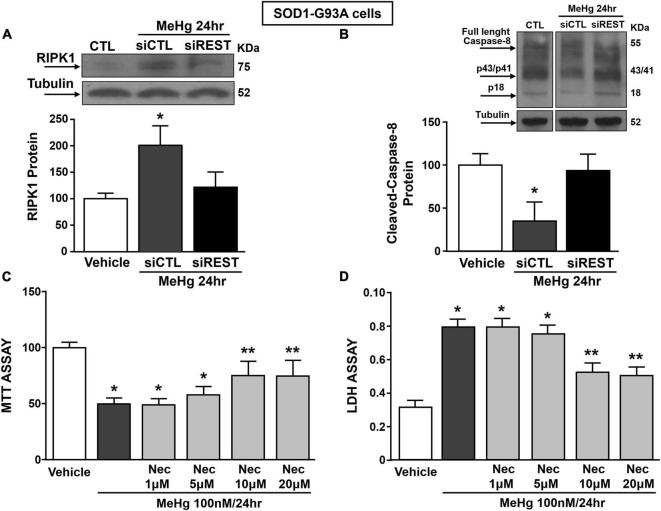
Effect of: (1) REST knocking-down on RIPK1 and cleaved-caspase 8 protein levels and (2) Necrostatin-1 (Nec) on neurosurvival in SOD1-G93A motor neuron-like NSC34 cells exposed to MeHg (100 nM) at 24 h. **(A,B)** Western Blot of REST in MeHg-exposed SOD1-G93A cells transfected with siREST. Graph shows the quantification of the ratio of RIPK1 and cleaved-caspase 8 to tubulin. Bars represent mean ± SD (*n* = 3) per group. *p ≤ 0.05 versus Veh, *#p* ≤ 0.05 versus siCTL + MeHg 24 h. **(C,D)** MTT assay and LDH release in SOD1-G93A cells pre-treated for 2 h with Nec at 1, 5, 10, and 20 μM and with MeHg (100 nM) for 24 h. Bars represent mean ± SD (*n* = 3). *p ≤ 0.05 versus Veh, **p ≤ 0.05 versus MeHg, MeHg + Nec 1 μM, MeHg + Nec 5 μM.

It has been previously demonstrated that necroptosis contributes to motor neuron death in amyotrophic lateral sclerosis (Yuan et al., 2019) and that the neurotoxicant PCB-95 activates the necroptotic pathway in cortical neurons *via* REST up-regulation (Guida et al., 2017b). It is widely documented that for the activation of necroptosis two conditions must be satisfied: (1) the activation of Receptor InterActing Serine/Threonine Kinase 1 (RIPK1) and (2) the blockade of caspase 8 activation. To this aim, we evaluated the effect of REST knocking-down on RIPK1 protein expression and caspase-8 activation in MeHg-treated SOD1-G93A cells. Notably, siREST: (1) counteracted the MeHg-mediated RIPK1 increase and (2) induced the activation of the caspase 8, thus preventing the activation of the necroptotic pathways (Figures 7A,B). Importantly, MTT and LDH assays demonstrated that the pre-treatment with the specific inhibitor of necroptosis Necrostatin 1 (Nec) (1, 5, 10, and 20 μM) determined in MeHg-treated SOD1-G93A cells a significant reduction in cell death (Figures 7C,D).

## Discussion

Our results indicate that MeHg determined necroptotic cell death in motor neuron-like SOD1-G93A cells by increasing the binding of the epigenetic complex Sp1/KMT2A on the promoter sequence of the transcriptional repressor REST, thus up-regulating its mRNA and protein levels. Several studies demonstrated the possible relationship between methylmercury exposure and ALS pathophysiology (Bailey et al., 2017). Interestingly, it has been demonstrated that the molecular mechanisms of G93A-induced cell death after MeHg exposure is related to glutamate release that activates α-amino-3-hydroxy-5-methyl-4-isoxazolepropionic acid receptor (AMPA) receptors, which in turn increases intracellular calcium concentrations and determines motor neuron neurotoxicity (Johnson and Atchison, 2009; Johnson et al., 2011). Interestingly, AMPA receptor (AMPAR) GluA2 subunit (gria2) is a REST target gene and is involved in stroke-induced neuronal death (Noh et al., 2012). Although glutamate neurotoxicity is widely endorsed by the scientific community as one of the main mechanisms in the ALS pathophysiology (Vucic et al., 2014) and REST is an important transcription factor regulating several glutamate receptor subunits (Rodenas-Ruano et al., 2012), our *in vitro* model could not be used to evaluate glutamate neurotoxicity, since it has been demonstrated that, even if differentiated, NSC34 cells do not express glutamate receptors (Madji Hounoum et al., 2016). However, we could not exclude that, by using another *in vitro* or *in vivo* models of ALS, REST could induce motor-neuronal cell death by triggering glutamate-mediated excitotoxicity. Therefore, the aim of our work is to evaluate the role of REST in inducing necroptotic neuronal death after methylmercury exposure and not by activating glutamate receptors.

Herein, we found that toxic concentrations of MeHg accelerate cell death in motor neuron-like NSC34 cells transfected with the SOD1-G93A construct *via* REST up-regulation, but not in NSC34 cells transfected with the SOD1-WT or empty vector. Furthermore, REST up-regulation is associated to neuronal death, indeed the knocking-down of REST by siRNA transfection reduced MeHg-induced neurodetrimental effect in SOD1-G93A motor neuron-like cells. These results are in accordance with previous studies demonstrating the neurotoxic role of REST after MeHg and PCBs exposures (Guida et al., 2017b,^2018^). MeHg-induced REST transcriptional activation in SOD1-G93A motor neuron-like cells is due to the activation of the transcriptional factor Sp1 that was the protein responsible of the REST mRNA and protein increase. It is interesting to mention that in another neurodegenerative disorder, such as Huntington disease, it has been demonstrated that in NG108 cells overexpressing mutant Huntingtin (mHtt) Sp1 up-regulates REST by binding its promoter sequence (Ravache et al., 2010). In addition, we recently showed that in cortical neurons REST gene is regulated by the transcriptional factors Sp1, Sp3, CREB, and JunD (Formisano et al., 2014; Guida et al., 2017b), whereas in the present study MeHg increased Sp1 mRNA and protein levels, but not CREB, Sp3 and JunD mRNA levels. Interestingly, the results of the present study demonstrated that Sp1 activates REST transcription by binding the alternative A region of the promoter sequence, in accordance with other study demonstrating that Sp1 binds REST A promoter sequence, but not the B and C regions in NG108 cells and mouse brain (Ravache et al., 2010). Furthermore, in the present study ChIP experiments performed in SOD1-G93A motor neuron-like cells demonstrated that MeHg promotes the interaction between the transcriptional factor Sp1 and the epigenetic writer KMT2A and that Sp1 silencing prevented KMT2A binding and H3K4-me3 accumulation on REST promoter sequence. These results suggest that Sp1 physically interacted with KMT2A forming a complex on REST A promoter sequence that produces the REST transcriptional activation. These data are supported by the fact that siKMT2A reverted MeHg-increased REST mRNA and protein levels in SOD1-G93A cells. The involvement of the epigenetic writer KMT2A in the molecular mechanisms elicited by MeHg is of particular interest since it has been demonstrated that it promotes neuronal apoptosis in the ischemic penumbra in an *in vivo* model of brain ischemia (Feng et al., 2020). In addition, we found that necroptosis was the cell death mechanism induced by REST after MeHg exposure, as revealed by the increase of the necroptotic marker RIPK1 and by the reduction of cleaved caspase-8 expression. More relevant, REST silencing significantly counteracted MeHg-induced RIPK1 increase and caspase-8 inactivation, suggesting that MeHg exposure by increasing REST modulates transcription of necroptosis pathway genes. These results are consistent with our previous study demonstrating that the neurotoxicants PCB-95 induced necroptotic cell death in cortical neurons by increasing REST expression and that caspase-8 is a REST gene target (Guida et al., 2017b). Necroptotic cell death mechanism has been identified as a relevant neurotoxic pathway in *in vitro* and *in vivo* models of ALS and the RIPK1- specific inhibitor Necrostatin-1 (Nec) was found to increase cell survival in both ALS models (Yuan et al., 2019). Herein, it has been demonstrated that MeHg-induced necroptotic cell death was reverted by Nec in a dose dependent manner and that Nec at the concentration of 10 μM displayed the maximum protective effect to reduce neuronal death in SOD1-G93A motor neuron-like cells. Undifferentiated state of NSC-34 cells transfected with EV, SOD1 and SOD1-G93A constructs are adequate cell model to study methylmercury (MeHg) neurotoxicity. Indeed, the neurotoxic effect of MeHg is mediated by variations in intracellular calcium concentrations (Limke et al., 2003), and since Chen P. C. et al. (2018) showed that retinoic acid-induced differentiation in NSC-34 motoneuronal-like cells exerts a suppressive effect on the expression and activity of Ca^2+^-activated K^+^ channels 3.1 (KCa3.1), thus regulating calcium homeostasis, the use of differentiated NSC-34 cells could reduce the sensitivity to MeHg neurotoxic stimulus. Furthermore, the differentiation state of NSC-34 cells is not critical to study the neuronal death mechanisms related to MeHg exposure since both differentiated and undifferentiated NSC-34 cells are equally sensitive to methylmercury neurotoxicity (Chapman and Chan, 1999). In addition, it should be underlined that both undifferentiated and differentiated NSC-34 cells transfected with SOD1-G93A vector display a similar reduction of cell survival (Oosthuyse et al., 2001; Turner et al., 2005; Grad et al., 2014). Collectively, these results demonstrate that motor neuron-like NSC34 cells overexpressing human SOD1-G93A protein are more sensitive to MeHg neurotoxicity, compared to cells overexpressing the wild type form of human SOD1. Furthermore, the findings of this research highlight that the transcriptional activator Sp1, by recruiting the epigenetic modifier KMT2A, up-regulates REST mRNA and protein levels in SOD1-G93A cells increasing the H3K4-me3 levels on REST gene promoter sequence. Finally, we found that the necroptosis was the cell death mechanism by which REST reduced cell survival in SOD1-G93A cells. Therefore, the discovery of KMT2A antagonists able to reduce H3K4-me3 expression, and thus to reduce REST levels, could represent a new and additional pharmacological strategy to intervene in the treatment of ALS patients.

## Data Availability Statement

The raw data supporting the conclusions of this article will be made available by the authors, without undue reservation.

## Author Contributions

NG contributed to conception, design, analysis of PCR experiments, and interpretation of data and manuscript writing. LS and LM performed and analyzed LDH, MTT, and Western Blot experiments. LC, AS, and PM designed and performed ChIP experiments. LA, LF, and GL contributed to financial support, conception, and design of all the experiments and manuscript writing. All authors contributed to the article and approved the submitted version.

## Conflict of Interest

The authors declare that the research was conducted in the absence of any commercial or financial relationships that could be construed as a potential conflict of interest.

## Publisher’s Note

All claims expressed in this article are solely those of the authors and do not necessarily represent those of their affiliated organizations, or those of the publisher, the editors and the reviewers. Any product that may be evaluated in this article, or claim that may be made by its manufacturer, is not guaranteed or endorsed by the publisher.

## Supplementary Material

Supplementary Figure 1 Effect of MeHg (0.1, 10, and 100 nM) at 24, 48, and 72 h on survival in motor neuron-like NSC34 EVcells. **(A–C)** MTT assay in motor neuron-like NSC34 EVcells exposed to MeHg 0.1, 1, and 100 nM at 24, 48 and 72 h. Bars represent mean ± SD (*n* = 3); **p* ≤ 0.05 versus vehicle (Veh).

Supplementary Figure 2 LDH assay at 24, 48, and 72 h in motor neuron-like NSC34 cells transiently transfected with the empty vector, SOD1-WT and SOD1-G93A constructs alone or in combination with MeHg (100 nM). **(A–C)** Effects of EV, SOD1-WT and SOD1-G93A vectors on cell survival, measured by LDH assay after 24, 48 and 72 h transfection. Bars represent mean ± SD (*n* = 3); **p* ≤ 0.05 versus EV and SOD1-WT; **(D–F)** Effect of 24, 48 and 72 h of MeHg (100 nM) exposure on LDH release in: **(A)** EV, **(B)** SOD1-WT and **(C)** SOD1-G93A cells. Bars represent mean ± SD (*n* = 3); **p* ≤ 0.05 versus vehicle (Veh); ^**^*p* ≤ 0.05 versus all.

Supplementary Figure 3 Experiments representing transfection efficiency of siREST and positive controls of antibodies used to investigate proteins expression. **(A)** qRT-PCR of REST in NSC34 cells transfected with siREST. Graph shows the quantification of the ratio of REST to β-Actin. Bars represent mean ± SD (*n* = 3). **p* ≤ 0.05 versus siCTL. **(B–G)** Western Blot in SH-SY5Y and NSC34 cells for REST, Sp1, H3K4-me3 and caspase-8.
