# Supplementary figures and images for: The Transcriptional Complex Sp1/KMT2A by Up-Regulating Restrictive Element 1 Silencing Transcription Factor Accelerates Methylmercury-Induced Cell Death in Motor Neuron-Like NSC34 Cells Overexpressing SOD1-G93A
Source: Front Neurosci. 2021 Nov 26;15:771580. doi: 10.3389/fnins.2021.771580 (PMC8662822; doi:10.3389/fnins.2021.771580)

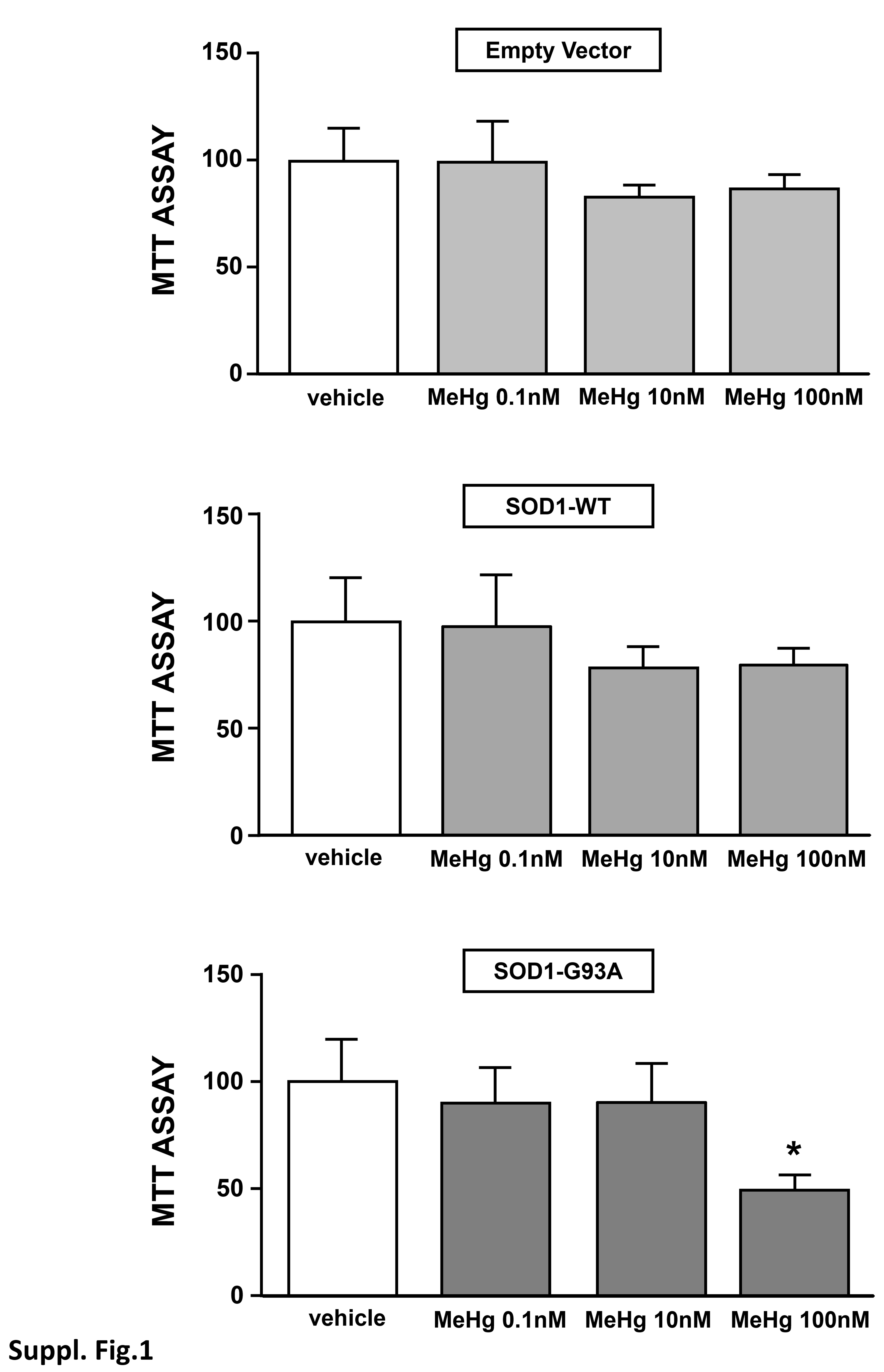

Supplement: Supplementary Figure 1 — Effect of MeHg (0.1, 10, and 100 nM) at 24, 48, and 72 h on survival in motor neuron-like NSC34 EVcells. (A–C) MTT assay in motor neuron-like NSC34 EVcells exposed to MeHg 0.1, 1, and 100 nM at 24, 48 and 72 h. Bars represent mean ± SD (n = 3); *p ≤ 0.05 versus vehicle (Veh). [file Image_1.tif]

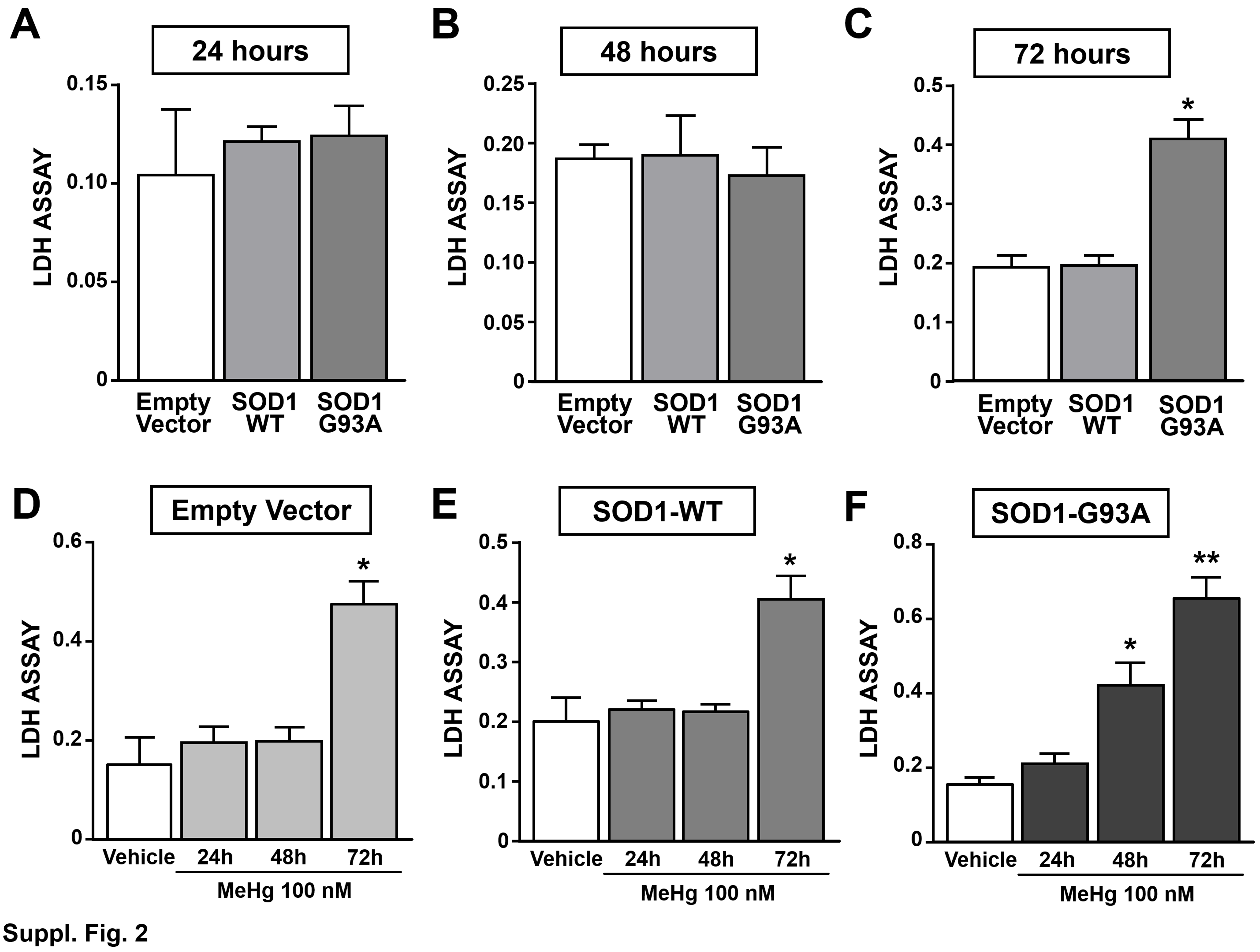

Supplement: Supplementary Figure 2 — LDH assay at 24, 48, and 72 h in motor neuron-like NSC34 cells transiently transfected with the empty vector, SOD1-WT and SOD1-G93A constructs alone or in combination with MeHg (100 nM). (A–C) Effects of EV, SOD1-WT and SOD1-G93A vectors on cell survival, measured by LDH assay after 24, 48 and 72 h transfection. Bars represent mean ± SD (n = 3); *p ≤ 0.05 versus EV and SOD1-WT; (D–F) Effect of 24, 48 and 72 h of MeHg (100 nM) exposure on LDH release in: (A) EV, (B) SOD1-WT and (C) SOD1-G93A cells. Bars represent mean ± SD (n = 3); *p ≤ 0.05 versus vehicle (Veh); **p ≤ 0.05 versus all. [file Image_2.tif]

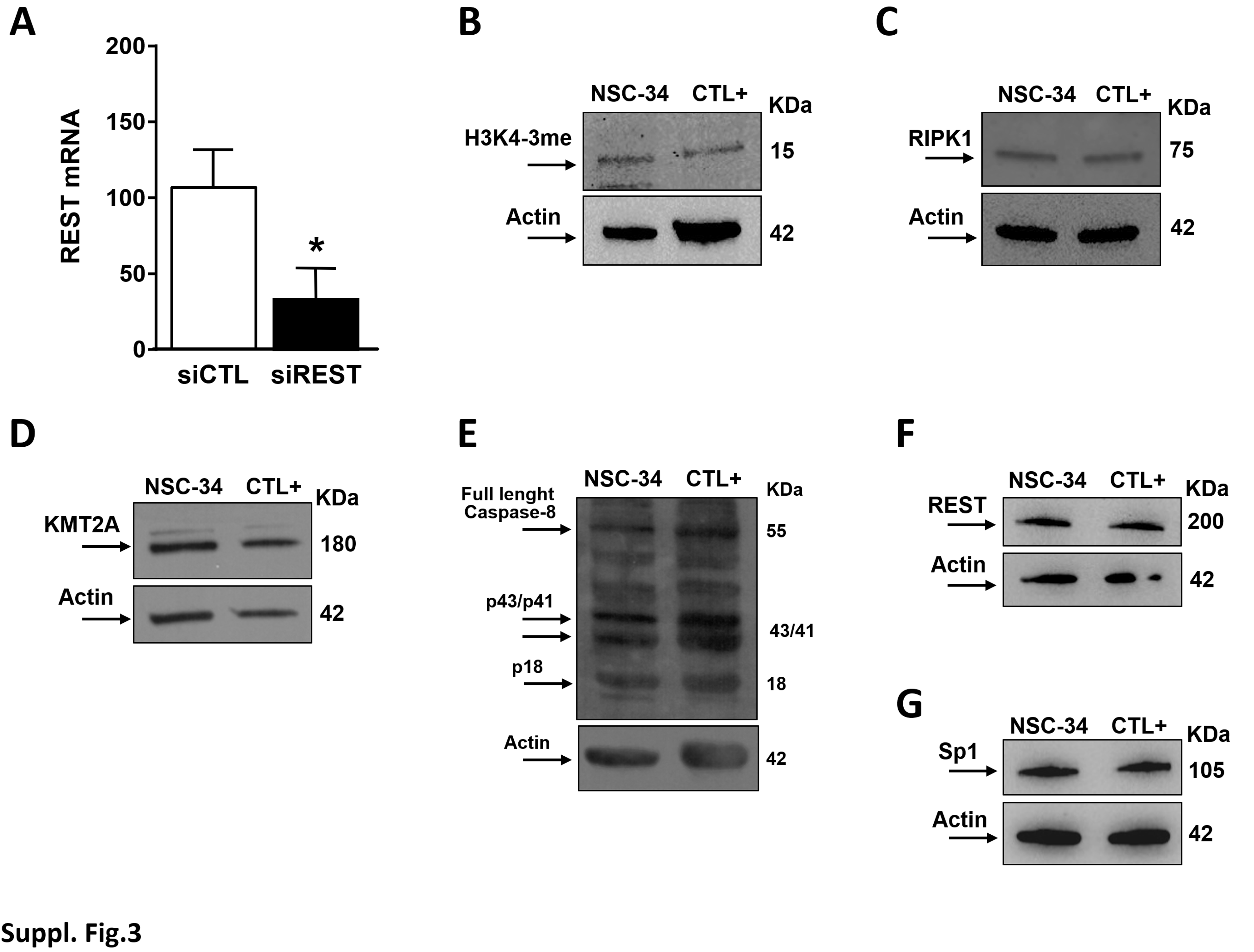

Supplement: Supplementary Figure 3 — Experiments representing transfection efficiency of siREST and positive controls of antibodies used to investigate proteins expression. (A) qRT-PCR of REST in NSC34 cells transfected with siREST. Graph shows the quantification of the ratio of REST to β-Actin. Bars represent mean ± SD (n = 3). *p ≤ 0.05 versus siCTL. (B–G) Western Blot in SH-SY5Y and NSC34 cells for REST, Sp1, H3K4-me3 and caspase-8. [file Image_3.tif]
